# Quantification of gene-specific methylation of *DNMT3B* and *MTHFR* using sequenom EpiTYPER®

**DOI:** 10.1016/j.dib.2015.11.039

**Published:** 2015-11-26

**Authors:** Vikki Ho, Janet E. Ashbury, Sherryl Taylor, Stephen Vanner, Will D. King

**Affiliations:** a Department of Public Health Sciences, Queen’s University Kingston, Ontario, Canada K7L3N6; b Department of Medical Genetics, University of Alberta, Edmonton, Alberta, Canada T6G 2R3; c Gastrointestinal Diseases Research Unit (GIDRU), Queen’s University, Kingston, Ontario, Canada K7L3N6

## Abstract

Among 272 patients undergoing a screening colonoscopy, DNA methylation of *DNMT3B* and *MTHFR*, genes encoding enzymes critical to one-carbon metabolism, was quantified in blood leukocytes using Sequenom EpiTYPER®. DNA methylation was quantified in 66 and 28 CpG sites of *DNMT3B* and *MTHFR* respectively, and conceptualized using two approaches. First, measures representing average methylation across all CpG sites were created. Second, unsupervised principal component (PC) analysis was used as a pattern derivation and data-reduction approach, to develop two summary variables (PC1 and PC2). These two summary variables represented methylation around the transcription start site (PC1) and in the gene-coding area (PC2) for both *DNMT3B* and *MTHFR*. The data contained in this article presents the variation of methylation levels for individual CpG sites within the *DNMT3B* and *MTHFR* genes and possible correlations uncovered using PC analysis. The data are related to the research article “Gene-specific DNA methylation of DNMT3B and MTHFR and colorectal adenoma risk” in Mutation Research – Fundamental and Molecular Mechanisms of Mutagenesis.

## **Specifications table**

**Table:** 

Subject area	Epidemiology
More specific subject area	Cancer, Epigenetics
Type of data	Table, figure
How data was acquired	Sequenom EpiTYPER
Data format	Raw data
Experimental factors	Leukocytic DNA was isolated from blood samples collected from 272 patients undergoing a screening colonoscopy. Ninety participants were diagnosed with one or more pathologically-confirmed tubular, tubullo-villous or villous adenoma(s), while 182 patients had no abnormality identified during colonoscopy.
Experimental features	The Sequenom EpiTYPER® technology was used to quantify DNA methylation in 66 and 28 CpG sites of *DNMT3B* and *MTHFR*.
Data source location	Kingston, Ontario, Canada
Data accessibility	Data is available with this article

## **Value of the data**

• Using the Sequenom EpiTYPER® technology, we quantified methylation levels of 66 and 28 CpG sites located within the promoter regions of *DNMT3B* and *MTHFR*, respectively; methylation levels of these CpG sites were highly variable. Future etiological studies investigating the role of gene-specific DNA methylation in *DNMT3B* and *MTHFR* could explore different conceptualizations of methylation including principal component analysis.
• The methodology used in processing raw data values for methylation levels quantified using the Sequenom EpiTYPER® technology could be used as a benchmark for future research.
• The data derived from our study can be used to inform future investigations relating methylation levels within specific CpG sites to gene expression for *DNMT3B* and *MTHFR.*

## 1 Data

DNA methyltransferase 3B (*DNMT3B*: NM_006892.3) and methylenetetrahydrofolate reductase (*MTHFR*; NM_005957.4) are two key genes which encode enzymes critical to 1C metabolism, disruption of which has increasingly been implicated in colorectal cancer etiology [1], [2]. DNA methylation involves the covalent addition of a methyl group to cytosine residues that precede guanine (CpG) and is the most commonly studied epigenetic process [3]. In the present work, among 272 patients undergoing a screening colonoscopy, we present the data of leukocytic methylation levels quantified using the Sequenom EpiTYPER® technology for 66 and 28 CpG sites in the *DNMT3B* and *MTHFR* genes, respectively. The data are related to the research article “Gene-specific DNA methylation of DNMT3B and MTHFR and colorectal adenoma risk” in Mutation Research – Fundamental and Molecular Mechanisms of Mutagenesis [4].

## 2 Experimental design, materials and methods

### 2.1 Overview of study design

Among the 330 patients, aged 40–65, undergoing a screening colonoscopy at Hotel Dieu Hospital in Kingston, Ontario, Canada, 272 participants were selected for this research as they met the minimum blood leukocyte DNA concentration required for DNA methylation analysis. Ninety participants were diagnosed with one or more pathologically-confirmed tubular, tubullo-villous or villous adenoma(s) while 182 patients had no abnormality identified during colonoscopy.

At the colonoscopy visit, a fasting blood sample was collected in a 10 ml ethylenediaminetetraacetic acid vacutainer. The vacutainer was placed immediately on ice and blood was centrifuged within 45 min; the buffy layer (blood leukocytes) was removed and stored at −20 °C. DNA was isolated from blood leukocytes and purified using the 5-PRIME DNA isolation kit (Inter Medico, Markham, ON, Canada). DNA concentrations were quantified using Quant-iT™ PicoGreen®, and DNA was stored at −20 °C until methylation analysis.

### 2.2 Quantification of gene-specific DNA methylation

DNA methylation of *DNMT3B* and *MTHFR* genes was quantified in stored leukocytic DNA using the Sequenom EpiTYPER® technology. EpiTYPER® uses base-specific cleavage and matrix-assisted laser desorption/ionization-time of flight mass spectrometry to quantify DNA methylation [5], [6], [7]. This validated and reproducible method enables a highly precise and quantitative measurement of methylation at multiple CpG sites within large promoter regions of genes [6], [7].

Methylation analysis was carried out at the McGill University and Génome Quebec Innovation Centre, Montreal, Quebec, Canada. Using Sequenom’s EpiDesigner^TM^ software, PCR primers were designed for the two genes of interest (Table 1). Specifically, the genomic region of interest for *DNMT3B* spanned 1977 base pairs (GRCh37/hg19: chr20: 31349361-31351338, positive strand) and was chosen from the promoter region based on the reported location of a CpG island and literature showing variability between cancer cell lines [8], [9], [10]. The region of interest for *MTHFR* spanned 2115 base pairs (GRCh37/hg19: chr1:11866473-11864358, negative strand) and included coverage over the promoter region and CpG island; as well, CpG sites located apart from the island approximately 1000 base pairs from the promoter region were also targeted [10], [11], [12], [13], [14].

Participant DNA samples were bisulfite-treated and purified using the EZ DNA Methylation-Gold kit from ZymoResearch (cat# D-5007) according to manufacturer’s protocol. This bisulfite conversion (BSC) step selectively deaminates unmethylated cytosines to uracil, but has no effect on 5-methyl-cytosines thereby leading to a primary DNA sequence change that permits differentiation of unmethylated cytosines from methylated cytosines.

The regions of interest for *DNMT3B* and *MTHFR* were analyzed in 4 and 3 fragments spanning approximately 500 base pairs each, respectively. For each of the 7 fragments, 25 ng of BSC DNA was used to quantify methylation ratios within CpG units (a unit consists of either an individual CpG site or aggregates of multiple CpG sites) located within each fragment. A methylation ratio equals the percentage of methylated cytosines at a specific CpG unit of a gene, divided by the total number of copies of that CpG unit in the sample. For CpG units that consisted of multiple CpG sites, the methylation ratio of that CpG unit was assigned to each of the CpG sites within that unit. Three 96-well plates containing participant samples were run per fragment and for quality control, two high methylated human DNA controls and two low methylated human DNA controls manufactured by EpigenDx were included on each plate. Reliability was assessed using the two high methylated quality control samples and coefficients of variation (CV) were calculated between-plates; the low methylated DNA quality control sample was not utilized as the CV is sensitive to small changes to the mean when the mean is close to zero. For the high methylated DNA quality control sample, the CV between-plates was 5%.

### 2.3 Raw data processing and statistical analysis

**Fig. 1:**
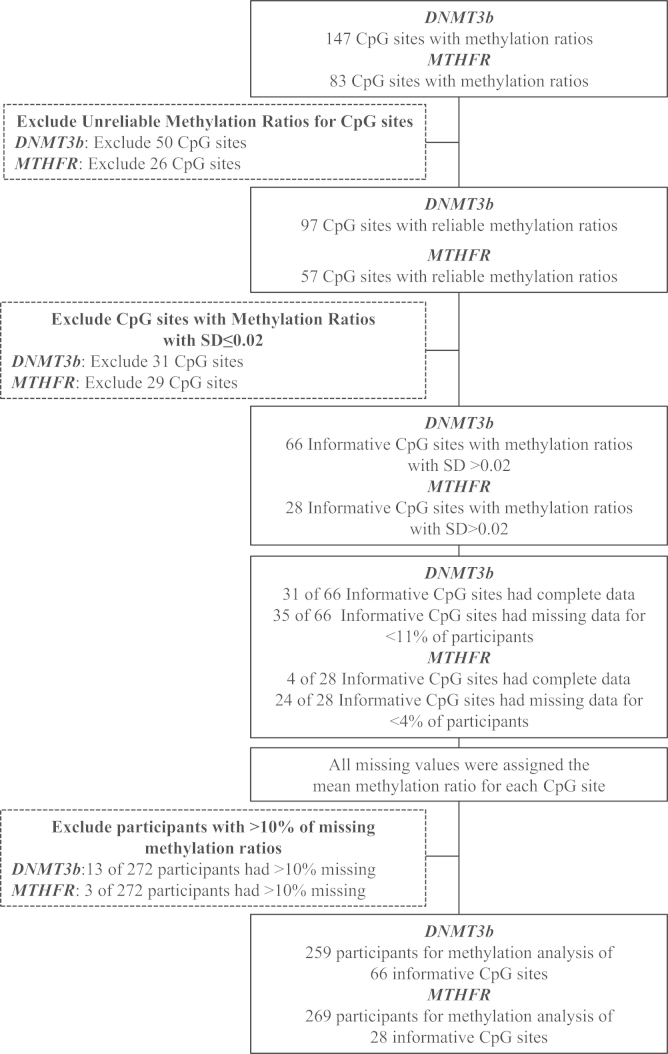
Data cleaning and processing of methylation ratios for CpG sites within the *DNMT3B* and *MTHFR* genes.

Raw data consisted of methylation ratios for 147 CpG sites for *DNMT3B* and 83 CpG sites for *MTHFR*. Methylation data was cleaned separately for *DNMT3B* and *MTHFR* to remove methylation ratios for CpG sites that were unreliable and had little variability (SD≤0.02) (Fig. 1). Specifically, unreliable methylation ratios for CpG sites were defined as either having: (1) low or high mass outside of the limit of detection of the Sequenom EpiTYPER assay or; (2) more than one silent peak or; (3) overlapping signals or; (4) duplicate CpG units of the same mass. Applying these criteria for *DNMT3B*, 50 of 147 methylation ratios for CpG sites were excluded. For *MTHFR*, 26 of 83 methylation ratios for CpG sites were excluded. CpG sites were subsequently defined as informative if they had methylation ratios with standard deviations >0.02; this cut-off was applied to ensure that the calculated methlyation measures were based on methylation ratios for CpG sites displaying meaningful differences. For *DNMT3B*, methylation ratios for 66 CpG sites had a standard deviation greater than 0.02. For *MTHFR*, methylation ratios for 28 CpG sites had a standard deviation greater than 0.02. These CpG sites were henceforth considered informative for statistical analysis. For *DNMT3B*, 31 of 66 informative CpG sites had complete data; for the remaining 35 CpG sites, methylation ratios for 1 or more CpG sites were missing for less than 11% of participants. Of the 28 informative CpG sites in *MTHFR*, data was complete for 4 CpG sites; for the remaining 24 CpG sites, methylation ratios for 1 or more CpG sites were missing for less than 4% of participants. All missing values were assigned the mean methylation ratio for each CpG site. To ensure that each participant had only a small proportion of missing values imputed, participants with data missing for >10% of CpG sites within *DNMT3B* or *MTHFR* were excluded. Based on this cutoff, a total of 259 and 269 participants were included in the statistical analysis for *DNMT3B* and *MTHFR*, respectively.

For the statistical analysis, gene-specific DNA methylation was conceptualized using two approaches. First, measures representing average methylation across all informative CpG sites were created. Second, unsupervised principal component (PC) analysis was used as a pattern derivation and data-reduction approach, to develop two summary variables (PC1 and PC2) which together accounted for 32% and 42% of the variance of DNA methylation within informative CpG sites in *DNMT3B* and *MTHFR,* respectively.

### 2.4 Descriptive analysis of gene-specific DNA methylation in DNMT3B and MTHFR

**Fig. 2:**
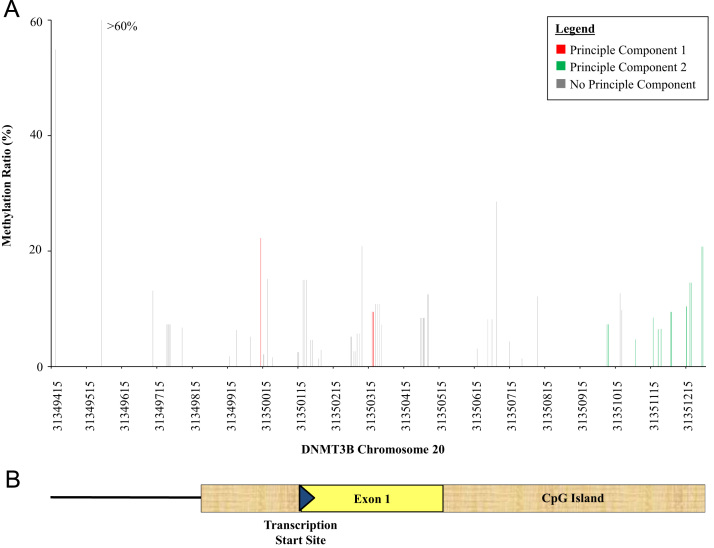
(A) Average methylation ratio for each informative CpG site in *DNMT3B* according to chromosome location. CpG sites with Eigenvalues greater than 0.20 for PC1 and PC2 are indicated in red and green, respectively. (B) Diagram illustrating the transcription start site and exon 1 of the *DNMT3b* gene within the targeted CpG island. (For interpretation of the references to color in this figure legend, the reader is referred to the web version of this article.)

**Fig. 3:**
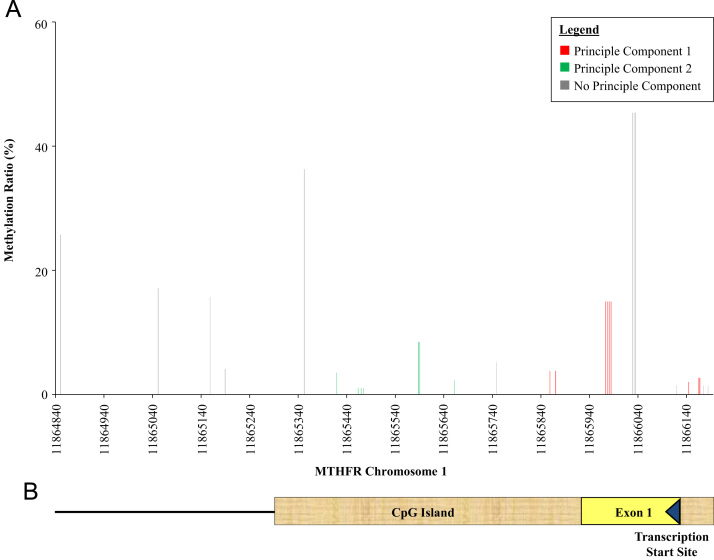
(A) Average percent methylation ratio for each informative CpG site in *MTHFR* according to chromosome location. CpG sites with Eigenvalues greater than 0.20 for PC1 and PC2 are indicated in red and green, respectively. (B) Diagram illustrating the transcription start site and exon 1 of the *MTHFR* gene within the targeted CpG island. (For interpretation of the references to color in this figure legend, the reader is referred to the web version of this article.)

The percent methylation of each informative CpG site for *DNMT3B* and *MTHFR* is presented in Fig. 2, Fig. 3*,* respectively. The *x*-axis of the figures denotes where each informative CpG site resides with reference to chromosome location; the *y*-axis denotes the percent methylation of each informative CpG site. For *DNMT3B*, average percent methylation across all informative CpG sites among events and non-events was 11% (SD=0.03) and 11% (SD=0.04), respectively. For *MTHFR*, average percent methylation among events was 13% (SD=0.03) while among non-events was 12% (SD=0.02).

To evaluate possible patterns and correlations within the data, we differentiated CpG sites which correlated with each PC. Specifically, in Fig. 2, Fig. 3, the CpG sites with Eigenvalues greater than 0.20 for PC1 are illustrated in red while those with Eigenvalues greater than 0.20 for PC2 are in green; CpG sites that did not have Eigenvalues greater than 0.20 for neither PCs are in gray. For both *DNMT3B* (Fig. 2) and MTHFR (Fig. 3), methylation ratios of CpG sites around the transcriptional start site and the gene-coding area were correlated and comprised PC1 and PC2.

## Figures and Tables

**Table 1:** Primer sequences for quantification of DNA methylation of *DNMT3B* and *MTHFR* genes using Sequenom EpiTYPER®. Upper case letters are used to denote the gene specific sequence in each primer shown.

Gene	Fragment	Region	Primer sequences	
**Forward 5′–3′**	**Reverse 5′–3′**
DNMT3b	1	chr20:31349428 to 31349886	aggaagagagTTGAGTATTTATTGAGATTTTGTTT	cagtaatacgactcactatagggagaaggctCAAACTCCTTCTAAAACCTTTTTCC
	2	chr20:31349921to 31350352	aggaagagagGGAAAAAGGTTTTAGAAGGAGTTTG	cagtaatacgactcactatagggagaaggctTTTACTTAAACCACTTAACCCCAAC
	3	chr20:31350385 to 31350876	aggaagagagGTTGGGGTTAAGTGGTTTAAGTA	cagtaatacgactcactatagggagaaggctCCTTAACTTTTCCCAAAACAAAAAC
	4	chr20:31350991 to 31351263	aggaagagagGTTTTTGTTTTGGGAAAAGTTAAGG	cagtaatacgactcactatagggagaaggctCTAAAAATAAAAAATAAAACCCCAA
				
MTHFR	1	chr1:11866175 to 11865734	aggaagagagGAGTTTTGGGATTGAGATTAGGAGT	cagtaatacgactcactatagggagaaggctAAACAAAAAACCAAAATCAATCTTC
	2	chr1:11865705 to 11865291	aggaagagagGAAGATTGATTTTGGTTTTTTGTTT	cagtaatacgactcactatagggagaaggctCAACTAAACTCCCATAACACCCTAA
	3	chr1:11865198 to 11864850	aggaagagagTAGGGTGTTATGGGAGTTTAGTTGA	cagtaatacgactcactatagggagaaggctAAAATTCATTTCTTTCAAACTATCCA
